# Novel Aptamer-Based Small-Molecule Drug Screening Assay to Identify Potential Sclerostin Inhibitors against Osteoporosis

**DOI:** 10.3390/ijms22158320

**Published:** 2021-08-02

**Authors:** Chien-Ching Lee, Chao-Ming Hung, Chung-Hwan Chen, Yi-Chiang Hsu, Yuan-Pin Huang, Tsung-Bin Huang, Mon-Juan Lee

**Affiliations:** 1 Department of Anesthesia, An Nan Hospital, China Medical University, Tainan 70965, Taiwan; otzison@gmail.com; 2 Department of Medical Science Industries, Chang Jung Christian University, Tainan 71101, Taiwan; 3 Department of General Surgery, E-Da Cancer Hospital, I-Shou University, Kaohsiung 82445, Taiwan; Ed100647@edah.org.tw; 4 School of Medicine, I-Shou University, Kaohsiung 82445, Taiwan; jenway74@isu.edu.tw; 5 Department of Orthopedics, Kaohsiung Municipal Ta-Tung Hospital, Kaohsiung 80145, Taiwan; hwan@kmu.edu.tw; 6 Department of Orthopedics, Kaohsiung Medical University Hospital, Kaohsiung Medical University, Kaohsiung 80756, Taiwan; 7 Orthopaedic Research Center, Kaohsiung Medical University, Kaohsiung 80756, Taiwan; 8 Department of Orthopedics, College of Medicine, Kaohsiung Medical University, Kaohsiung 80756, Taiwan; 9 Regenerative Medicine and Cell Therapy Research Center, Kaohsiung Medical University, Kaohsiung 80756, Taiwan; 10 Department of Cosmetics and Fashion Styling, Cheng Shiu University, Kaohsiung 83347, Taiwan; henry@gcloud.csu.edu.tw; 11 Bachelor Degree Program in Orchid Industry Application, Chang Jung Christian University, Tainan 71101, Taiwan; binbin@mail.cjcu.edu.tw; 12 Department of Bioscience Technology, Chang Jung Christian University, Tainan 71101, Taiwan

**Keywords:** osteoporosis, sclerostin, aptamer, drug screening, competitive assay, bone formation, drug repurposing, lead identification

## Abstract

A novel aptamer-based competitive drug screening platform for osteoporosis was devised in which fluorescence-labeled, sclerostin-specific aptamers compete with compounds from selected chemical libraries for the binding of immobilized recombinant human sclerostin to achieve high-throughput screening for potential small-molecule sclerostin inhibitors and to facilitate drug repurposing and drug discovery. Of the 96 selected inhibitors and FDA-approved drugs, six were shown to result in a significant decrease in the fluorescence intensity of the aptamer, suggesting a higher affinity toward sclerostin compared with that of the aptamer. The targets of these potential sclerostin inhibitors were correlated to lipid or bone metabolism, and several of the compounds have already been shown to be potential osteogenic activators, indicating that the aptamer-based competitive drug screening assay offered a potentially reliable strategy for the discovery of target-specific new drugs. The six potential sclerostin inhibitors suppressed the level of both intracellular and/or extracellular sclerostin in mouse osteocyte IDG-SW3 and increased alkaline phosphatase activity in IDG-SW3 cells, human bone marrow-derived mesenchymal stem cells and human fetal osteoblasts hFOB1.19. Potential small-molecule drug candidates obtained in this study are expected to provide new therapeutics for osteoporosis as well as insights into the structure–activity relationship of sclerostin inhibitors for rational drug design.

## 1. Introduction

Osteoporosis is considered a major and global public health concern. Because osteoporosis occurs primarily as a result of normal ageing and menopausal estrogen deficiency, the prevalence of osteoporotic fractures is expected to rise as the proportion of older populations increases worldwide [1]. The major drug that stimulates bone formation is parathyroid hormone 1–34, PTH (1–34), which is approved by the U.S. Food and Drug Administration (FDA) to treat osteoporosis at high risk for fracture in both men and women [2]. However, it was shown that prolonged treatment of F344 rats with 30 μg/kg PTH (1–34) for 20 or 24 months increases the incidence of bone cancer [3]. Other therapeutic options for bone loss, such as bisphosphonates, are antiresorptive agents without bone-stimulating activities.

Sclerostin is a secreted glycoprotein expressed predominantly in osteocytes. It inhibits bone formation by antagonizing the Wnt/β-catenin signaling pathway, which plays an important role in regulating the proliferation and differentiation of osteoblasts, and is upregulated during mechanical unloading [4,5,6]. Sclerostin was first recognized in patients with sclerosteosis, in which inactivating mutations of the sclerostin gene *SOST* were found to be associated with high bone mass [7]. It is responsible for the inhibition of osteoblast differentiation as well as stimulation of RANKL from osteocytes to induce osteoclastogensis [8,9,10] and promotes the apoptosis of human mesenchymal stem cells [11]. Animal studies confirmed that sclerostin knockout mice displayed a high bone mass phenotype with increased bone formation and bone mineral density [12], while overexpression of sclerostin in mice resulted in osteopenia [13]. These results demonstrate that sclerostin is a key negative regulator of bone formation. Antagonizing sclerostin is therefore considered a new strategy for the treatment of osteoporosis [5]. Romosozumab, a humanized anti-sclerostin monoclonal antibody designed for subcutaneous administration, was approved for the treatment of severe osteoporosis and postmenopausal women at high risk for osteoporotic fracture [14,15]. Results from a phase III clinical trial indicate that romosozumab gave rise to higher bone mineral density (BMD) in postmenopausal women higher than placebo or PTH (1–34) [16,17] and significantly reduced fracture risks compared to treatment with placebo or oral alendronate [17,18]. Although romosozumab was demonstrated in clinical studies as a promising therapeutic for osteoporosis, increasing number of severe cardiovascular adverse events posed a potential risk to patients with cardiovascular diseases, and the safety and side effects of long-term treatment are unclear [19].

Aptamers are synthetic, single-stranded DNA or RNA molecules isolated from combinatorial oligonucleotide libraries using an in vitro selection method called SELEX (systematic evolution of ligands by exponential enrichment) [20,21]. Aptamers with three-dimensional structure and high affinity for various molecular targets, such as small molecules, proteins, nucleic acids and even cells, tissues and organisms, can thus be produced efficiently. Compared to antibodies, aptamers can be synthesized rapidly and cost-efficiently, are compatible with various labeling and detection strategies, and are versatile in specific binding to a wide array of targets, in addition to proteins. Aptamers are not only considered artificial substitutes for antibodies but also novel therapeutics and ligands for target validation and lead identification in high-throughput screening (HTS) [22]. Over the past few decades, the structure-activity relationship between chemical compounds and their target proteins was derived from structural and functional genomics studies, which greatly improved the process of lead identification and optimization. Nevertheless, this route to drug discovery is limited by the availability of high-resolution protein structures and the software or tools to study a structure–activity relationship. Aptamers fill in the gap by presenting the complementary structural information of the target in their three-dimensional structures, so that small-molecule ligands to protein targets can be identified and optimized, even in the absence of complete information on protein structure, enzymatic or ligand-binding properties.

Green et al. were the first to design a competitive assay with a radioactive 27-nucleotide ssDNA aptamer against platelet-derived growth factor B-chain (PDGF-BB) [23]. Binding affinities of the PDGF-BB inhibitors are correlated with their inhibitory potencies in functional assays. Hartig et al., extended the competitive assay to an HTS-compatible format by constructing “aptazyme”, a hybrid RNA molecule comprising the catalytic core of the hammerhead ribozyme and an anti-Rev aptamer [24]. A structurally diverse library of antibiotics was screened with this platform for novel inhibitors of HIV replication. The binding affinity of aptamers is usually unaffected by conjugation with detection molecules such as fluorescent dyes, and labeling is not necessary for the target protein or the small-molecule drug candidates. These aptamer-based assay systems therefore offer new and powerful molecular biology tools for target validation and lead identification in drug discovery [22,25].

In this study, an aptamer-based competitive assay was established to screen for potential small-molecule sclerostin inhibitors and to facilitate drug repurposing by producing reliable hits from currently available chemical libraries. Drug repurposing, the process of discovering new therapeutic uses for existing drug candidates, is considered to shorten the time required to identify new drug candidates and increase the productivity of the pharmaceutical industry [26]. As low-molecular-weight, synthetic chemicals dominate the pharmaceutical market, the vast amount of chemicals synthesized and studied has culminated to various chemical libraries and databases. The development of efficient HTS assays has therefore become a crucial step to accelerate drug repurposing and drug discovery. Because aptamers are characterized by specific binding and antagonistic effects on protein function [25], sclerostin is a suitable target for competitive drug screening using sclerostin-specific aptamers. Chemical compounds with affinity comparable to that of an aptamer are expected to be potent inhibitors of sclerostin. The idea of a competitive assay is not new, but has not been fully explored in aptamer-based drug screening. We propose to establish an aptamer-based competitive assay in which the fluorescence-labeled, sclerostin-specific aptamers compete with compounds from selected chemical libraries for binding to immobilized recombinant human sclerostin to achieve high-throughput screening for potential small-molecule sclerostin inhibitors.

## 2. Results

### 2.1. Construction of the Chemical Library for Aptamer-Based Drug Screening

Studies on biomaterials designed to induce osteogenic differentiation found that the functional groups exposed to human mesenchymal stem cells may control their differentiation fate [6,27,28,29]. Although an absolute correlation has not been concluded, it was shown that the amino group is one of the functional groups to promote osteogenic differentiation [6,29]. Compounds with tertiary amino groups may be protonated at neutral pH of the cell culture medium to provide a biochemical and electrical environment favorable to osteogenesis. As our first attempt in drug screening for sclerostin inhibitors, 96 compounds containing primary, secondary or tertiary amino groups were selected from the commercially available inhibitor library and FDA-approved drug library. Of the 96 selected chemicals, 40 are FDA-approved drugs, 42 contain at least one tertiary amino group, 35 have primary and/or secondary amino groups but not tertiary ones, and 19 exhibit no amino group (Supplementary Table S1).

### 2.2. Aptamer-Based Competitive Drug Screening Assay

**Figure 1:**
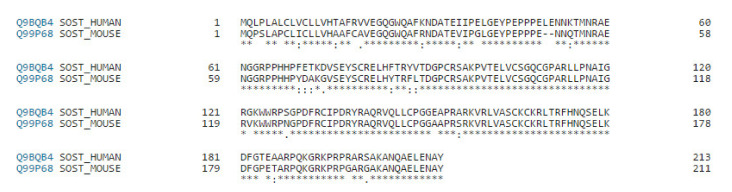
Amino acid sequence alignment of human and mouse sclerostin. Multiple sequence alignment was performed online by CLUSTAL Omega v.1.2.4 (http://www.clustal.org/omega/, accessed on 26 May 2017).

**Figure 2:**
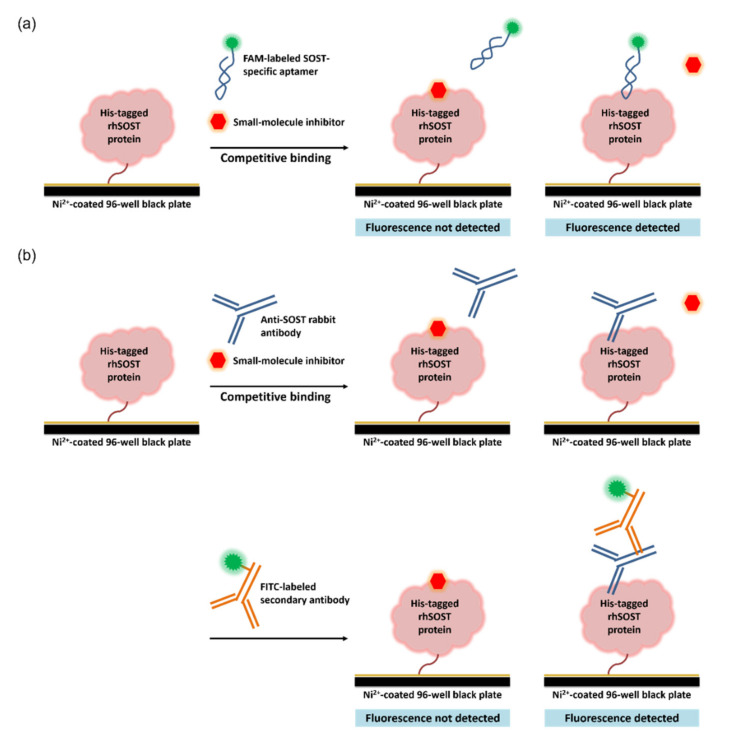
Aptamer-based and antibody-based competitive assays for small-molecule drug screening. The assay platforms were constructed by immobilizing His-tagged recombinant human sclerostin on a nickel-coated surface, followed by reacting with small-molecule compounds as potential drug candidates in the presence of (**a**) FAM-labeled sclerostin-specific aptamer or (**b**) anti-sclerostin antibody followed by detection with FITC-labeled secondary antibodies.

Because the amino acid sequence of mouse sclerostin and human sclerostin share 86.9% identity (Figure 1), a DNA aptamer against mouse sclerostin identified by Shum et al. was applied in the design of the aptamer-based competitive drug screening assay [30]. As shown in Figure 2a, immobilization of sclerostin in the 96-well black plate relied on the binding affinity between the nickel (Ni^2+^) coating on the plate surface and the His-tagged recombinant human sclerostin protein. The Ni^2+^-coated plate was pre-blocked with bovine serum albumin (BSA), allowing for the elimination of BSA blocking after the binding reaction. Ni^2+^-based affinity binding is sensitive to metal chelating agents such as EDTA and reducing agents such as mercaptoethanol, but is compatible with nonionic detergents and dimethyl sulfoxide (DMSO), in which stock solutions of the chemical compounds were prepared. The density of immobilized sclerostin protein on the Ni^2+^-coated surface was determined according to the manufacturer’s instruction, which indicates that the binding capacity of the nickel-coated 96-well plate is ~9 pmol/well, corresponding to ~200 ng/well His-tagged sclerostin protein. The immobilized human sclerostin was then reacted with 500 nM FAM-labeled sclerostin-specific aptamer in the presence of potential drug candidates.

**Figure 3:**
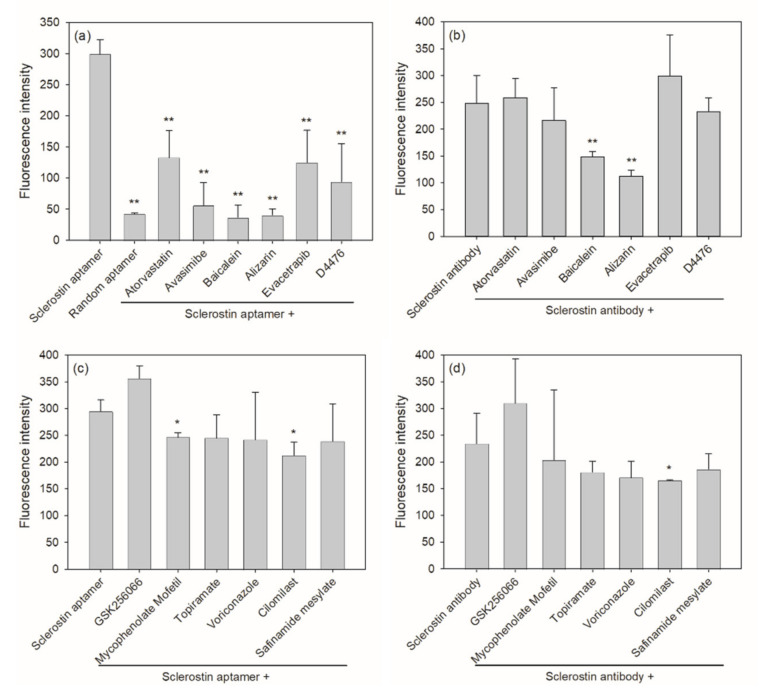
Fluorescence intensity of the sclerostin-specific aptamer or the sclerostin antigen–antibody immunocomplex in the presence of selected small-molecule inhibitors. His-tagged sclerostin protein was immobilized at 200 ng/well in a nickel-coated 96-well black plate and reacted with (**a**,**c**) 500 nM FAM-labeled sclerostin-specific aptamer in the presence or absence of 20 μM of one of the inhibitors, or (**b**,**d**) 0.52 μg/mL anti-sclerostin antibody (dilution ratio 1:500) in the presence or absence of 200 μM of one of the inhibitors, followed by detection with FITC-labeled secondary antibodies. Fluorescence intensity was determined by a TECAN Infinite 200 PRO F Nano^+^ microplate reader at Ex485 nm/Em520 nm. Error bars represent standard deviations (*n* ≥ 3). * *p* < 0.05 and ** *p* < 0.01, compared to the control.

It is assumed that compounds with binding affinity close to or higher than that of the aptamer may associate with the target protein, thus displacing the fluorescence-labeled aptamer. Potential inhibitors of sclerostin can then be identified by a significant decrease in fluorescence compared to that of the aptamer-only control. In the presence of 20 μM small-molecule inhibitors, 6 of the 96 selected inhibitors resulted in a significant decrease in fluorescence to more than 50% of the aptamer-only control (Figure 3a and Table 1). Results from the aptamer-based drug screening were compared to those of an antibody-based competitive immunoassay, in which the six selected small-molecule inhibitors competed with anti-sclerostin antibodies for binding to the recombinant sclerostin protein (Figure 2b). A higher concentration (200 μM) of the chemical library was used in the antibody-based competitive drug screening because at 20 μM, the reduction in fluorescence caused by the six potential sclerostin inhibitors was insignificant. As shown in Figure 3b, two of the six chemical compounds listed in Table 1, baicalein and alizarin, were found to significantly reduce the fluorescence intensity of the sclerostin antigen‒antibody immunocomplex, although the decrease in fluorescence (40 and 55% relative to the control for baicalein and alizarin, respectively) was less significant than that observed in the aptamer-based competitive drug screening assay (88 and 86% relative to the control for baicalein and alizarin, respectively). The less significant reduction in fluorescence and the higher concentration of the chemical library required in the antibody-based competitive assay may be explained at least in part by the higher affinity of the antibody (*K*_D_ usually in the nanomolar range) toward the sclerostin protein, compared to that of the aptamer (*K*_D_ = 0.67 ± 0.08 μM according to [30]). The lower molecular weight of the 30-mer DNA aptamer (9.5 kDa) compared to the 150-kDa anti-sclerostin antibody may also lead to easier replacement by small-molecule compounds, making aptamers a more suitable “competitor” in low-molecular-weight drug screening. Representative results of six other chemicals which did not cause over 50% reduction in fluorescence in the aptamer-based competitive drug screening assay are shown in Figure 3c for comparison, with the corresponding results from the antibody-based competitive assay in Figure 3d.

**Figure 4:**
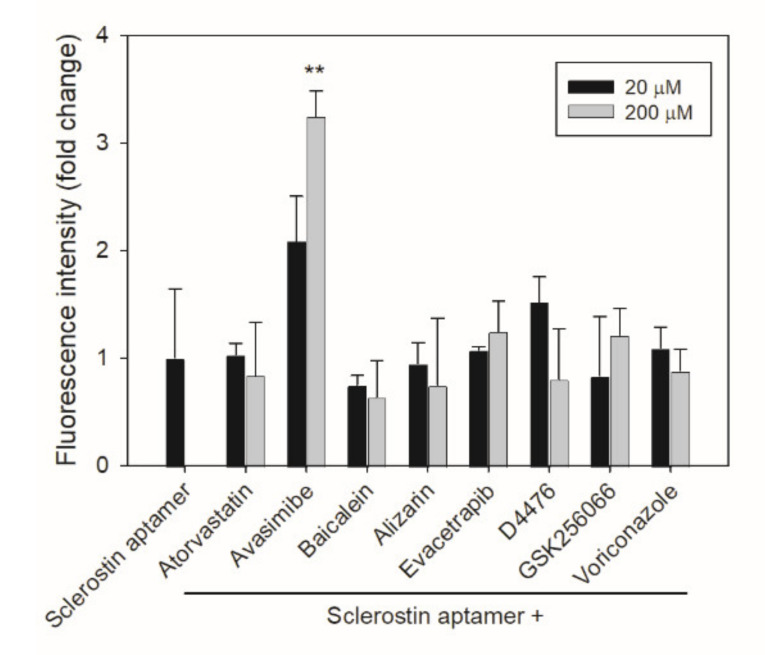
Fluorescence quenching effect of the potential sclerostin inhibitors. Each chemical compound was mixed with 500 nM FAM-labeld sclerostin aptamer at 20 or 200 μM in a nickel-coated 96-well black plate in the absence of immobilized sclerostin for 1 h. Fluorescence intensity was determined by a TECAN Infinite 200 PRO F Nano^+^ microplate reader at Ex485 nm/Em520 nm. Error bars represent standard deviations (*n* ≥ 3). ** *p* < 0.01, compared to the control.

We then investigated whether fluorescence quenching was responsible for the reduction of fluorescence observed in Figure 3a,b.The aptamer-based competitive drug screening assay was performed as described previously but without the immobilized sclerostin. When 20 or 200 μM of the chemical compound was incubated with 500 nM FAM-labeled sclerostin aptamer, a slight decrease in fluorescence intensity was observed for baicalein (Figure 4). Although alizarin absorbs at the emission wavelength of FAM (520 nm), the quenching effect of alizarin was not significant at both 20 and 200 μM. On the other hand, avasimibe enhanced the florescence of FAM at both 20 and 200 μM. Nevertheless, these conditions are not comparable to the actual experimental setting during fluorescence detection. When competitive drug screening was performed in the presence of immobilized sclerostin, the unbound aptamer and chemical compound were washed away prior to fluorescence detection. Unless the chemical compound was tightly associated with or in close proximity to the FAM-labeled aptamer, the amount of residual baicalein or alizarin in the final solution should be much lower than 20 μM for fluorescence quenching to occur. Furthermore, in the antibody-based competitive drug screening assay, unbound anti-sclerostin antibodies and the small-molecule inhibitors were washed away before the FITC-labeled secondary antibody was added, at which stage the residual chemical compound should be bound to sclerostin (Figure 2b). As the possibility of interaction between unbound baicalein or alizarin and the FITC-labeled secondary antibody is relatively unlikely, the reduction in fluorescence can be attributed primarily to the displacement of anti-sclerostin antibodies by baicalein or alizarin.

### 2.3. Intracellular and Extracellular Levels of Sclerostin in Mouse IDG-SW3 Cells

**Figure 5:**
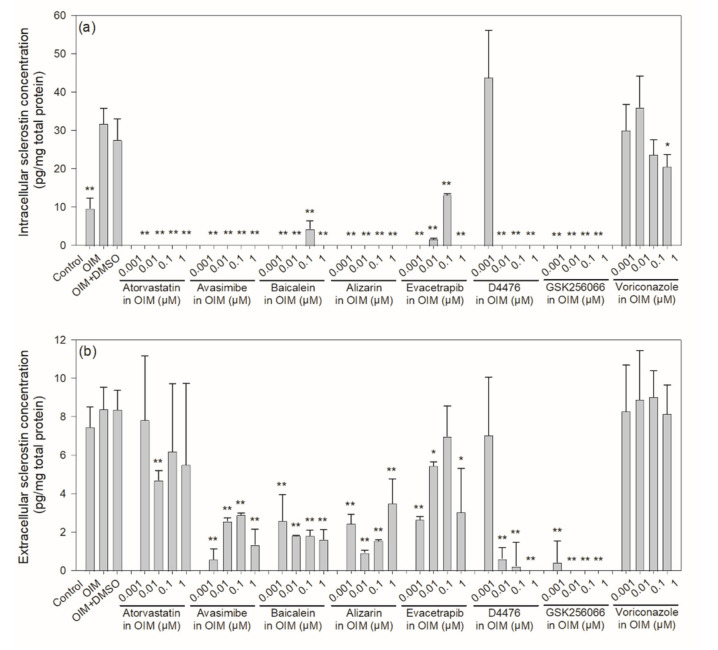
Intracellular and extracellular levels of sclerostin in IDG-SW3 cells treated with various concentrations of potential sclerostin-binding inhibitors. IDG-SW3 cells were treated with 0.001‒1 μM of one of the 6 potential sclerostin inhibitors, as well as the negative controls GSK256066 and voriconazole, in OIM for 21 days, followed by collection of the cell lysate and conditioned medium to determine the (**a**) intracellular and (**b**) extracellular sclerostin concentration, respectively, with a mouse sclerostin ELISA kit. The concentration of sclerostin was expressed as the amount of sclerostin (pg) normalized to total protein (mg) in the cell lysate or conditioned medium. Error bars represent standard deviations (*n* ≥ 3). * *p* < 0.05 and ** *p* < 0.01 compared to the OIM controls.

Physiologically, sclerostin is secreted by mature, embedded osteocytes, but according to the literature, existing osteocyte cell lines lack the expression of sclerostin [31]. In order to study the effect of the drug candidates on sclerostin expression, the mouse osteoblast-to-late-osteocyte cell line IDG-SW3 was used [31]. As shown in Figure 5, when cultured in osteogenic induction medium (OIM) for 21 days, the level of intracellular sclerostin was increased to approximately three-fold of the control, while that of extracellular sclerostin remained similar to that of the control. In the presence of 0.001–1 μM of the six potential sclerostin inhibitors, intracellular sclerostin was almost depleted, while extracellular sclerostin decreased significantly at several or all concentrations tested compared to the untreated and OIM controls, especially for avasimibe, alizarin, baicalein and D4476. Although sclerostin was proven to be a crucial therapeutic target for skeletal diseases, its molecular mechanism of regulation and degradation remains unclear. In addition to transcriptional regulation through the sclerostin gene, a recent report has shown that rapid, minute-scale lysosomal degradation may also be responsible for its down-regulation [32]. Nevertheless, these results provide strong evidence that the six potential sclerostin-binding inhibitors effectively suppressed the level of both intracellular and extracellular sclerostin. Results for two other chemical compounds, GSK256066 and voriconazole, which did not cause a significant reduction in fluorescence in the aptamer-based and antibody-based competitive drug screening assays, as shown in Figure 3c,d, are also presented in Figure 5 for comparison. Voriconazole, an inhibitor of the P450 isoenzyme CYP2B6 and an antifungal medication, did not affect the protein expression of sclerostin intracellularly or extracellularly. In contrast, intracellular and extracellular levels of sclerostin were both significantly reduced in the presence of GSK256066, a phosphodiesterase 4 inhibitor.

### 2.4. Cellular Rescue Assay and Osteogenic Activity of Potential Sclerostin-Binding Inhibitors

**Figure 6:**
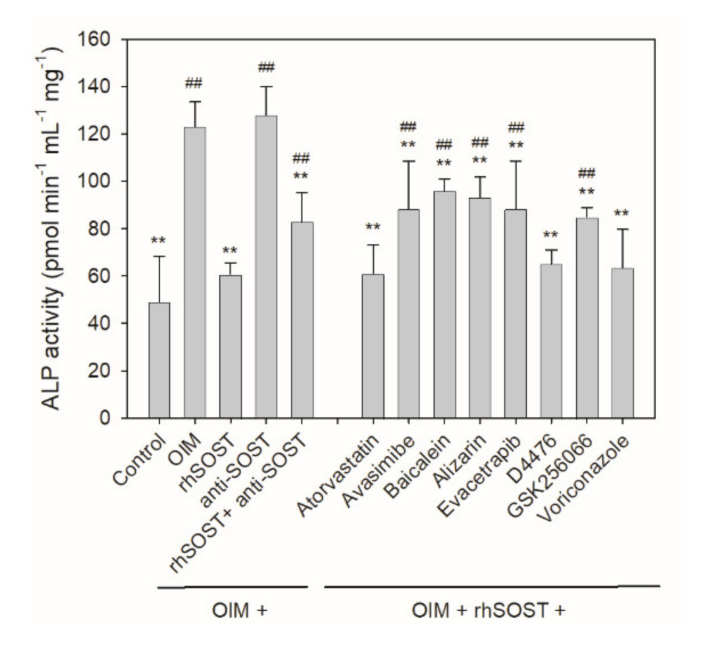
Effect of the potential sclerostin inhibitors on rescuing suppressed ALP activity caused by exogenous sclerostin. Recombinant human sclerostin (rhSOST) was incubated with 1 μM of the chemical compound for 30 min prior to incubation with hBMSCs for 3 days. After refreshing the medium with OIM, the cells were cultured for another 4 days before ALP activity was determined. Error bars represent standard deviations (*n* ≥ 3). ^##^ *p* < 0.01, compared to the control; ** *p* < 0.01, compared to the OIM control.

To examine whether the sclerostin-binding effect of potential sclerostin inhibitors observed in the drug screening assay was valid at the cellular level, a rescue assay was designed by pre-incubating recombinant human sclerostin (rhSOST) with anti-sclerostin rabbit antibody or selected small-molecule inhibitors under osteogenic induction conditions at room temperature for 30 min prior to culturing with human bone marrow-derived mesenchymal stem cells (hBMSCs) for three days. After culturing in OIM for another four days, the result of the rescue assay was assessed by measuring alkaline phosphatase (ALP) activity, an early marker for osteoblast maturation. As shown in Figure 6, ALP activity was completely abrogated in the presence of 10 μg/mL rhSOST (rhSOST vs. control and OIM), but remained unaffected at 1 μg/mL rhSOST (data not shown). Treatment with 10 μg/mL anti-sclerostin antibody did not further enhance ALP activity because secreted sclerostin is undetectable in vitro in hBMSC culture in the early stage of osteogenic differentiation for sclerostin neutralization to take place (anti-SOST vs. OIM in Figure 6). However, in the presence of exogenous rhSOST, the suppressed ALP activity was partially alleviated by co-incubating with the anti-sclerostin antibody (rhSOST + anti-SOST vs. control and OIM in Figure 6). The partial recovery in ALP activity was also observed when small-molecule inhibitors such as avasimibe, baicalein, alizarin, evacetrapib or GSK256066 were present (Figure 6).

**Figure 7:**
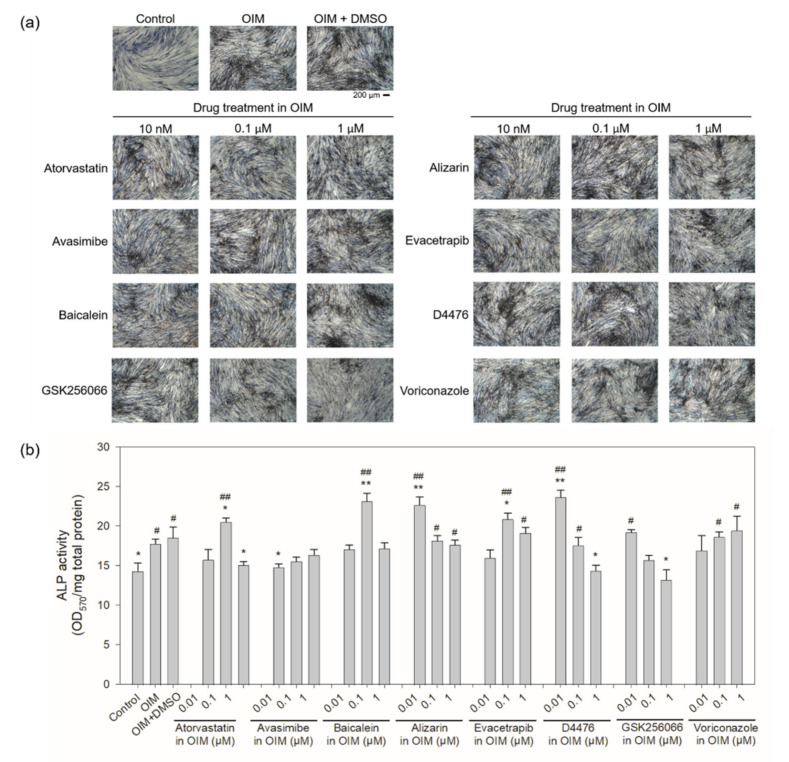
Effect of potential sclerostin-binding inhibitors on the ALP activity of hBMSCs. (**a**) Human BMSCs were cultured in OIM in the presence of various concentrations of potential sclerostin inhibitors, as well as the negative controls GSK256066 and voriconazole, for 21 days, followed by cell staining with Pierce 1-Step NBT/BCIP Solution (Thermo Fisher Scientific, Waltham, MA, USA) according to the manufacturer’s instructions. (**b**) The level of alkaline phosphatase activity was quantitated by solubilizing the cells in DMSO and determining the optical density at 570 nm, which was then normalized to total protein (mg). Error bars represent standard deviations (*n* ≥ 3). ^#^ *p* < 0.05 and ^##^ *p* < 0.01, compared to the control; * *p* < 0.05 and ** *p* < 0.01, compared to the OIM controls.

**Figure 8:**
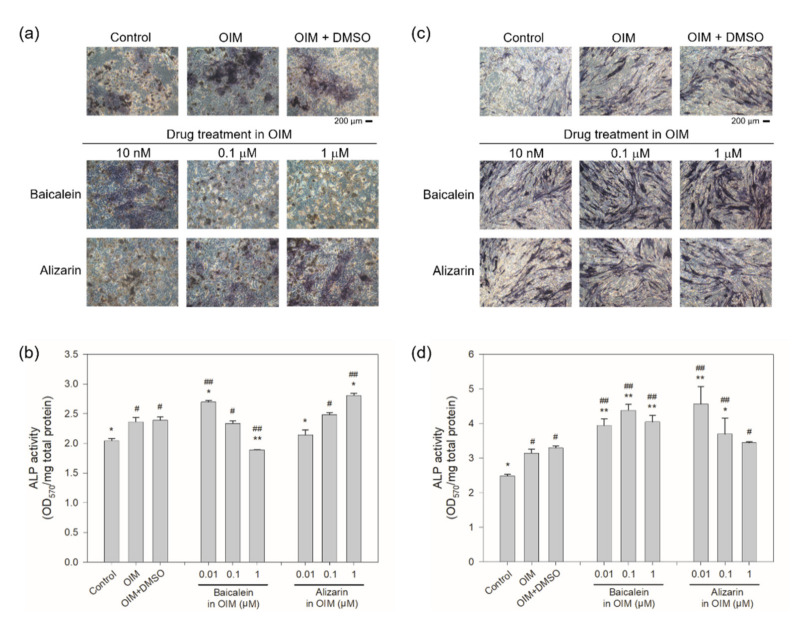
Effect of baicalein and alizarin on the ALP activity of IDG-SW3 and hFOB1.19 cells. (**a**,**c**) Mouse IDG-SW3 and human hFOB1.19 cells were treated with OIM in the presence of various concentrations of baicalein or alizarin for 14 days, followed by cell staining with Pierce 1-Step NBT/BCIP Solution (Thermo Fisher Scientific, Waltham, MA, USA) according to the manufacturer’s instructions. (**b**,**d**) The level of alkaline phosphatase activity was quantitated by solubilizing the cells in DMSO and determining the optical density at 570 nm, which was then normalized to total protein (mg). Error bars represent standard deviations (*n* ≥ 3). ^#^ *p* < 0.05 and ^##^ *p* < 0.01, compared to the control; * *p* < 0.05 and ** *p* < 0.01, compared to the OIM controls.

To further demonstrate whether the ALP activity was enhanced by potential sclerostin-binding inhibitors in the rescue assay, hBMSCs were cultured for 21 days in OIM containing the inhibitors to analyze their osteogenic activity. Enhanced ALP activity was found at 0.1 μM atorvastatin, baicalein or evacetrapib and at 0.01 μM alizarin or D4476 in hBMSCs (Figure 7). In comparison, no significant change in ALP activity was observed in the presence of voriconazole, while GSK256066 down-regulated ALP activity in a dose-dependent manner. When IDG-SW3 was cultured in OIM with baicalein and alizarin, the two chemical compounds that resulted in the most significant decrease in fluorescence in both aptamer-based and antibody-based drug screening, the ALP activity of the mouse osteocyte was significantly higher than that of the control and OIM-treated cells at 10 nM baicalein, but decreased with increasing concentrations of baicalein, while that in the presence of alizarin increased dose-dependently (Figure 8a,b). On the other hand, higher ALP activity was detected in hFOB1.19 cells compared to the OIM controls for both baicalein and alizarin (Figure 8c,d). As opposed to sclerostin-specific aptamer and antibody, whose effect was mediated extracellularly through neutralization of secreted sclerostin, the small-molecule inhibitors may enter the cells and exert their effect both intracellularly and extracellularly. The accelerated osteogenic differentiation observed in IDG-SW3 cells in the presence of baicalein or alizarin may be associated with the suppression of intracellular and extracellular sclerostin (Figure 5). However, as the protein expression of sclerostin was barely detectable in hBMSCs and hFOB1.19 cells (data not shown), due possibly to the late expression of sclerostin in the osteogenic differentiation process of osteoblast to osteocyte, the direct association of sclerostin suppression to osteogenic differentiation in hBMSCs and hFOB1.19 cells remains to be investigated. The enhanced ALP activity observed in hBMSCs and hFOB1.19 therefore suggests that in addition to interacting directly with intracellular and extracellular sclerostin, the potential sclerostin inhibitors may act through other pathways as well to stimulate bone formation. The cellular assays, including the analysis of sclerostin protein level (Figure 5) and ALP activity assay (Figure 6, Figure 7 and Figure 8), may serve as preliminary in vitro validations to assess the osteogenic potential of the drug candidates in relation to sclerostin.

## 3. Discussion

The small-molecule inhibitors were initially selected from the chemical libraries and categorized based on the amino group, because studies have shown that it is one of the functional groups to promote osteogenic differentiation. The functional group served only as an initial criterion for selecting compounds from the vast database of the chemical libraries, and a correlation between the amino group and the affinity to sclerostin was not assumed or observed in our drug screening results. Among the six potential sclerostin inhibitors, atorvastatin contains a secondary and a tertiary amino group, evacetrapib has two tertiary amino groups and D4476 has a primary and a secondary amino group, while no amino group is present in avasimibe, baicalein and alizarin. Although the binding site of the sclerostin-specific aptamer or antibody remains unclear, the NMR structure of both human and mouse sclerostin revealed surface areas populated with positively charged amino acids to interact with the negatively charged heparin or the negatively charged region of human low-density lipoprotein receptor-related protein 5 (LRP5), respectively [33,34]. The highly positively charged surface of sclerostin may not favor the interaction of compounds with protonated tertiary amino groups, but could provide binding sites for those with carboxyl groups such as atorvastatin and evacetrapib. In addition, the hydrophobic patch found on human sclerostin may be considered another site for the binding of relatively nonpolar or uncharged ligands [33].

Because the conditions for competitive drug screening do not correspond to the actual physiological condition, we analyzed the effect of the potential sclerostin inhibitors on cellular sclerostin level and ALP activity as further validation. Although it was assumed that chemical compounds which compete with the aptamer or antibody for binding to sclerostin may be considered potential sclerostin inhibitors, this does not necessarily imply that those that do not interact directly with sclerostin have no effect on its expression or exhibit no osteogenic activity, as compounds selected for drug screening are known protein or enzyme inhibitors and may act on various pathways to regulate sclerostin and the Wnt signaling pathways. For the two selected negative controls, voriconazole did not affect the protein expression of sclerostin intracellularly or extracellularly, and no significant change in the ALP activity of hBMSCs was observed in the presence of voriconazole. In contrast, intracellular and extracellular levels of sclerostin were both significantly reduced, but ALP activity was also down-regulated in a dose-dependent manner in the presence of GSK256066. Furthermore, the results from this study indicate that the fluorescence of FAM-labeled sclerostin-specific aptamer and FITC-conjugated anti-sclerostin antibody was not affected in the presence of icariin, but the small-molecule inhibitor was reported to promote the osteogenic differentiation of rat BMSCs through the regulation of sclerostin [35].

In the cellular assays, the sclerostin-specific DNA aptamer is susceptible to nuclease degradation in cell culture medium and has to be modified in order to enhance its stability [30], and was therefore not considered as the positive control. Despite the general understanding that the anti-sclerostin antibody promotes bone formation by neutralizing secreted sclerostin, it is not a suitable positive control either because the sclerostin protein bound by the antibody remained in the conditioned medium and can still be detected in ELISA by the immobilized capture antibody that recognizes a different epitope. In addition, the osteogenic effect of the anti-sclerostin antibody, although demonstrated in animal and clinical studies, cannot be easily observed in vitro in cells induced to differentiate osteogenically. In IDG-SW3 cells, which exhibit osteoblast to late osteocyte characteristics during osteogenic differentiation, both sclerostin expression and ALP activity were enhanced, according to the literature [31] and results from this study (Figure 5a and Figure 8a,b). It is therefore assumed that the osteogenic inducers, i.e., ascorbic acid and β-glycerophosphate supplemented in the osteogenic induction medium, overcome the antagonistic effect of secreted sclerostin, resulting in the up-regulation of ALP activity. The anti-sclerostin antibody added to IDG-SW3 cells may be continuously counteracted by secreted sclerostin, rendering its bone-stimulating effect insignificant. Moreover, the practice of refreshing the medium every three or four days may also eliminate in part the effect of accumulated sclerostin and anti-sclerostin antibodies. On the other hand, the amount of secreted sclerostin from hFOB1.19 and hBMSCs cultured under osteogenic conditions was too low for the anti-sclerostin antibody to exert its effect. It was reported that in hFOB1.19 cells, the extracellular sclerostin level was approximately 0.4 pg/mL [36], far lower than that detected in IDG-SW3 cells (~22 pg/mL in this study). For human mesenchymal stem cells, *SOST* gene expression was increased when cultured under osteogenesis-inducing conditions [37]. However, the extracellular level of the sclerostin protein was barely detectable in our study (data not shown).

In addition to its antagonistic effect on osteogenic differentiation, sclerostin is involved in the crosstalk between adipose and bone tissues [38]. Sclerostin promotes adipogenesis in mouse adipocyte 3T3-L1 and mouse BMSCs [39,40]. White adipose tissue was reduced in Sost^−/−^ mice, accompanied by an increase in bone mass, which can be explained by enhanced Wnt/β-catenin signaling in the absence of sclerostin, leading to the up-regulation of fatty acid catabolism and down-regulation of fatty acid synthesis [41]. It is therefore reasonable that several of the potential sclerostin inhibitors obtained from the drug screening assay are themselves inhibitors of enzymes involved in lipid metabolism. Of the six potential sclerostin inhibitors, atorvastatin calcium, an FDA-approved drug to lower low-density lipoprotein cholesterol in blood, is the inhibitor of HMG-CoA reductase, avasimibe, baicalein and alizarin are cytochrome P450 inhibitors, evacetrapib (LY2484595) inhibits cholesteryl ester transfer protein (CETP), while D4476 inhibits both casein kinase 1 (CK1) and TGFβ type I receptor kinase (ALK5). Both in vitro and in vivo studies support that statins, or HMG-CoA reductase inhibitors, promote osteogenesis and bone formation [42]. Atorvaststin was proven to enhance bone formation in apolipoprotein E-deficient (apoE^–/–^) mice by increasing the gene and protein expression of Sirt1 and Runx2 [43]. Although its association with bone formation was unclear, treatment of adipocytes with avasimibe resulted in significant reduction of lipid accumulation, which is the reciprocal effect of osteogenic differentiation in mesenchymal stem cells [44,45]. The osteogenic potential of baicalein has been reported in human periodontal ligament cells [46], but correlation to sclerostin has not been reported. Baicalein was also shown to increase callus density and cortical width, and may be beneficial to a later stage of the bone healing process [47]. Alizarin was generally used as the anthraquinone dye in matrix mineralization staining, but its osteogenic effect has not been discussed. Nevertheless, other anthraquinone compounds such as aloin and emodin, both of which are natural anthraquinones derived from plants, were shown to induce osteogenic differentiation [48,49]. Finally, targets of evacetrapib and D4476, CETP and ALK5 are related to lipid metabolism and osteogenesis, and inhibition of which led to an increased level of high-density lipoprotein cholesterol in clinical trials [50] or enhanced alkaline phosphatase activity in osteoblastic mesenchymal cells [51], respectively. As the targets of these potential sclerostin inhibitors all play important roles in lipid or bone metabolism, and several of the compounds have already been shown to be potential osteogenic activators, it is suggested that the aptamer-based competitive drug screening assay designed in this study offered a potentially reliable strategy for the discovery of target-specific new drugs.

## 4. Materials and Methods

### 4.1. Materials

Sclerostin-specific aptamer, 5′-TTGCGCGTTAATTGGGGGGGTGGGTGGGTT-3′ [30], labeled with 6-carboxyfluorescein (FAM) at the 3′ end, was synthesized by Integrated DNA Technologies, Inc, Coralville, IA, USA. *N*-terminally histidine (His)-tagged recombinant human sclerostin protein and rabbit anti-sclerostin antibody was provided by Abcam, Cambridge, MA, USA. Pierce nickel-coated 96-well black plate pre-blocked with BSA was purchased from Thermo Fisher Scientific, Waltham, MA, USA. FITC-labeled rabbit secondary antibody was manufactured by Santa Cruz Biotechnology, Dallas, TX, USA. A chemical library of selected small-molecule inhibitors was provided in 10 mM stock solutions in DMSO by Selleck Chemicals, Houston, TX, USA.

### 4.2. Aptamer-Based Competitive Drug Screening Assay

A working solution of His-tagged recombinant human sclerostin protein was freshly prepared at a concentration of 2 ng/μL in tris-buffered saline (TBS, 50 mM Tris-Cl, pH 7.5, and 150 mM NaCl) and was added at 100 μL/well to a nickel-coated 96-well black plate. After reaction at room temperature for 1 h, each well was washed 3 times with 200 μL/well TBS buffer. To perform the competitive assay, 500 nM FAM-labeled sclerostin-specific aptamer was prepared in TBS buffer and was heated at 95 °C for 5 min, followed by cooling to room temperature for 15 min to promote the formation of 3D structures. The FAM-labeled aptamer was added to the 96-well plate at 100 μL/well, followed by mixing with one selected compound per well from the chemical library to a final concentration of 20 μM. The mixture of FAM-labeled aptamer and selected chemical compound was then incubated with the immobilized sclerostin protein at room temperature for another hour. After washing 3 times with 200 μL/well TBS buffer, the fluorescence intensity of each well was determined by a TECAN Infinite 200 PRO F Nano^+^ microplate reader (Tecan Trading AG, Switzerland) at Ex485 nm/Em520 nm.

### 4.3. Antibody-Based Competitive Drug Screening Assay

As a comparative study, an anti-sclerostin rabbit antibody was substituted for the sclerostin-specific aptamer in the competitive assay. After immobilization of the His-tagged recombinant human sclerostin protein at 200 ng/well on the nickel-coated surface, 0.52 μg/mL anti-sclerostin rabbit antibody (dilution ratio 1:500) prepared in TBS buffer along with 200 μM of the selected chemical compound were added in a total volume of 100 μL/well. After reaction for 1 h at room temperature followed by washing 3 times with 200 μL/well TBS buffer, each well was incubated with 100 μL FITC-labeled rabbit secondary antibody (dilution ratio 1:100) for another 1 h. After washing 3 times with 200 μL/well TBS buffer, the fluorescence intensity was determined by a TECAN Infinite 200 PRO F Nano^+^ microplate reader at Ex485 nm/Em520 nm.

### 4.4. IDG-SW3 Cell Culture

Mouse osteoblast-to-late-osteocyte cell line IDG-SW3 (Kerafast, Inc., Boston, MA, USA) was seeded at a density of 4 × 10^4^ cells/cm^2^ and maintained at 33 °C and 5% CO_2_ in α-MEM supplemented with 8‒50 U/mL recombinant mouse interferon gamma (IFN-γ), 10% fetal bovine serum (FBS), 100 U/mL penicillin and 100 μg/mL streptomycin [31]. All culture dishes for IDG-SW3 cells were pre-coated with rat tail type I collagen and were products of Corning Inc., Corning, NY, USA, including Corning^®^ BioCoat™ Collagen I 100 mm TC-treated Culture Dishes and Corning^®^ BioCoat™ Collagen I Clear Flat Bottom TC-treated Multiwell Plates. Osteogenic differentiation of IDG-SW3 cells was induced at 37 °C and 8% CO_2_ by incubating the cells in growth medium supplemented with 50 μg/mL ascorbic acid and 4 mM β-glycerophosphate in the absence of IFN-γ.

### 4.5. hBMSC Culture

Poietics^TM^ Normal Human Bone Marrow Derived Mesenchymal Stem Cells (CD105^+^, CD166^+^, CD29^+^, CD44^+^, CD90^+^, CD73^+^, CD14^−^, CD34^−^, CD45^−^, CD19^−^ and HLA-DR-negative) were provided by Lonza, Basel, Switzerland and were isolated from normal (non-diabetic) adult human bone marrow withdrawn from bilateral punctures of the posterior iliac crests of normal volunteers. The cells were cultured at 37 °C and 5% CO_2_ with MSCGM^TM^ Mesenchymal Stem Cell Growth Medium BulletKit^TM^ (Lonza, Basel, Switzerland) according to the manufacturer’s instructions. The hBMSCs used in this study were cultures between passages 3 and 5. To induce osteogenic differentiation, hBMSCs were cultured for 14–21 days in OIM, which consists of DMEM supplemented with 10% FBS, 10^−7^ M dexamethasone, 10 mM β-glycerolphosphate, 50 μM L-ascorbate 2-phosphate, 100 U/mL penicillin and 100 μg/mL streptomycin.

### 4.6. hFOB1.19 Cell Culture

The human fetal osteoblast cell line hFOB1.19 was provided by the Bioresource Collection and Research Center of the Food Industry Research and Development Institute, Hsinchu, Taiwan. Cells were maintained at 33.5 °C in the presence of 5% CO_2_ in a growth medium consisting of a 1:1 mixture of DMEM and Ham’s F-12 medium in the absence of phenol red (Thermo Fisher Scientific, Waltham, MA, USA), with additional 2.5 mM L-glutamine, 0.3 mg/mL G418 and 10% FBS. Osteogenic induction was performed at 37 °C in the growth medium supplemented with 5 × 10^‒3^ mol/L β-glycerophosphate, 0.1 g/L ascorbic acid, 10^‒8^ mol/L menadione and 10^‒7^ mol/L 1,25-dihydroxyvitamin D_3_.

### 4.7. SOST ELISA

IDG-SW3 cells were treated with 0.001, 0.01, 0.1 or 1 μM of a potential sclerostin inhibitor under osteogenesis-inducing conditions for 21 days. The conditioned medium and cell lysate at day 21 were collected, and the concentration of intracellular and extracellular sclerostin was determined by a Mouse/Rat SOST/Sclerostin Quantikine ELISA Kit produced by R&D Systems, Minneapolis, MN, USA. The concentration of sclerostin was then normalized to the amount of total protein, which was quantitated by a Bio-Rad DC Protein Assay kit (Bio-Rad Laboratories, Inc., Hercules, CA, USA).

### 4.8. Cellular Rescue Assay

Human BMSCs were seeded at 3000 cells/well in a 96-well black plate with a flat clear bottom and cultured in growth medium for 3 days. To perform the cellular rescue assay, 10 μg/mL recombinant human sclerostin protein was pre-incubated with 10 μg/mL anti-sclerostin rabbit antibody or 1 μM of the chemical compound for 30 min prior to culturing with hBMSCs for 3 days. After refreshing the medium with OIM, the cells were cultured for another 4 days before ALP activity was determined by an Alkaline Phosphatase Activity Fluorometric Assay Kit manufactured by BioVision Inc., Milpitas, CA, USA.

### 4.9. Alkaline Phosphatase Activity Assay

Human BMSCs, IDG-SW3 and hFOB1.19 were seeded at 10^4^, 8 × 10^4^ and 10^4^ cells/well, respectively, in a 24-well plate and cultured in the presence of various concentrations of the potential sclerostin inhibitor prepared in OIM for 14 or 21 days. ALP activity was determined by fixing the cells with 4% paraformaldehyde in PBS for 15 min, followed by washing successively with PBS and ddH_2_O. The cells were then reacted with 300 μL/well Pierce 1-Step NBT/BCIP Solution (Thermo Fisher Scientific, Waltham, MA, USA) for approximately 30 min at room temperature so that the blue stain of the ALP reaction product can be seen with the naked eye. After washing again with ddH_2_O, the stained cells were air-dried before microscopic images were captured.

### 4.10. Statistical Analysis

Statistical significance was evaluated by Student’s *t* test. Levels of significance were expressed as significant, *p* < 0.05 and highly significant, *p* < 0.01, respectively.

## 5. Conclusions

Although romosozumab was recently approved as an effective therapeutic antibody drug for osteoporosis, the long-term health risk of antibody treatment remains to be evaluated. For a promising disease-related target such as sclerostin, alternative interventions such as small-molecule drugs are worth investigating, which may provide a broader selection of medication with various routes of administration to meet the diverse needs of patients with different degrees of bone loss. The common features in the chemical structure of baicalein and alizarin and their osteogenic potential demonstrated in this study suggest that flavonoids and anthraquinones such as baicalein and alizarin may serve as lead compounds for the development of bone-stimulating new drugs. With novel molecular biology tools such as aptamers, the process of lead identification is expected to be simplified and accelerated to find new cures for devastating diseases with limited therapeutic options, such as osteoporosis. Potential small-molecule drug candidates obtained in this study are expected to accelerate the development of new therapeutics as well as offer more insights into the structure–activity relationship of sclerostin inhibitors for rational drug design.

## Figures and Tables

**Table 1:** A list of potential drug candidates screened from 96 small-molecule inhibitors that resulted in more than 50% reduction in fluorescence intensity compared to the control in the aptamer-based competitive drug screening assay.

Product Name	M.W.	CAS Number	Chemical Structure ^2^	Target
Atorvastatin calcium ^1^	1155.34	134523-03-8	C_66_H_68_CaF_2_N_4_O_10_ 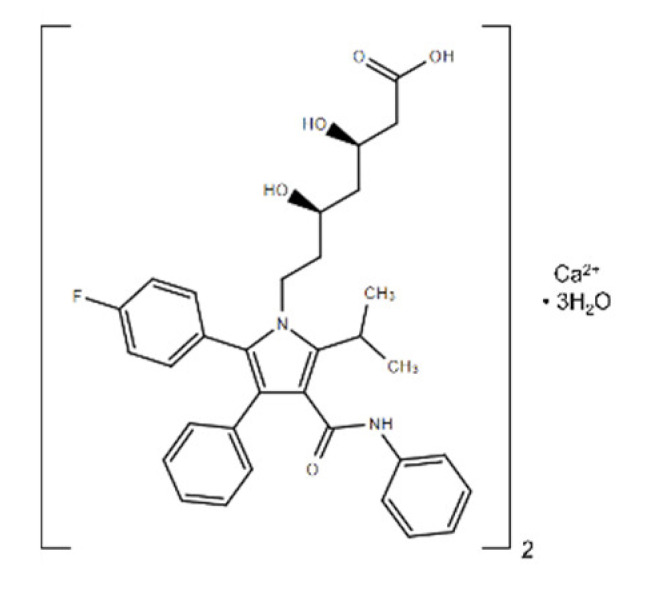	HMG-CoA reductase
Avasimibe	501.72	166518-60-1	C_29_H_43_NO_4_S 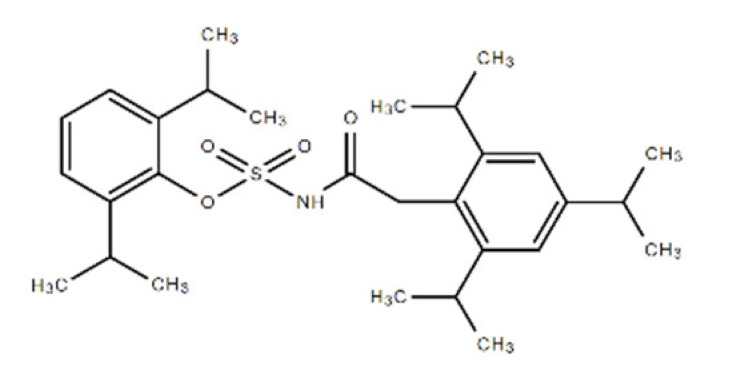	Cholesterol acyltransferase (ACAT) and P450 isoenzymes CYP2C9, CYP1A2 and CYP2C19
Baicalein	270.24	491-67-8	C_15_H_10_O_5_ 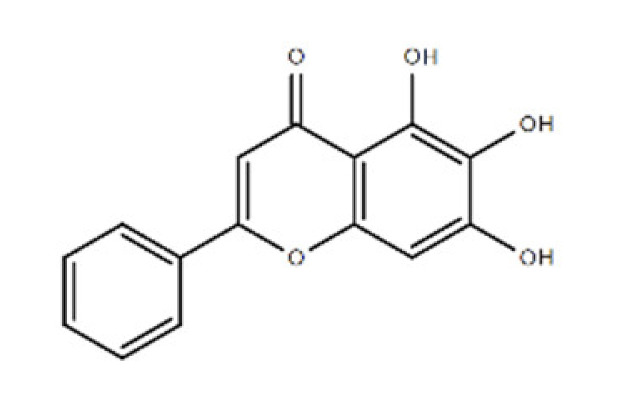	P450 isozyme CYP2C9 and prolyl endopeptidase
Alizarin	240.21	72-48-0	C_14_H_8_O_4_ 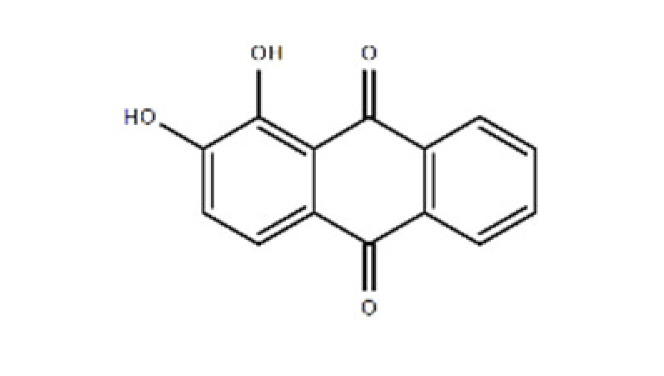	P450 isoforms CYP1A1, CYP1A2 and CYP1B1
Evacetrapib (LY2484595)	638.65	1186486-62-3	C_31_H_36_F_6_N_6_O_2_ 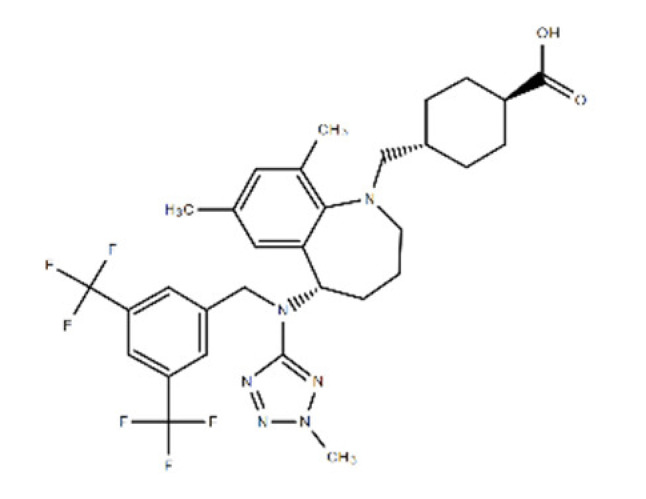	cholesteryl ester transfer protein (CETP)
D4476	398.41	301836-43-1	C_23_H_18_N_4_O_3_ 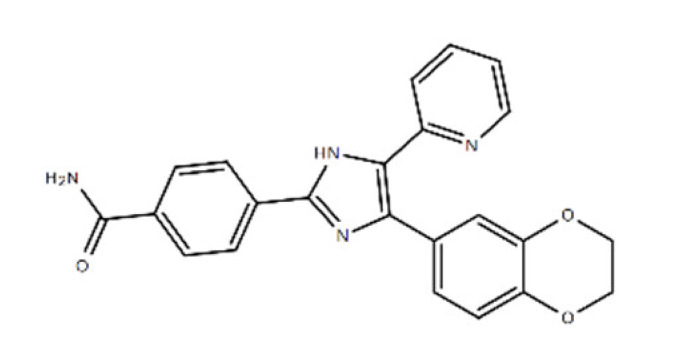	Casein kinase 1 (CK1) and TGFβ type I receptor kinase (ALK5)

^1^ FDA-approved drug; ^2^ The chemical structures were obtained from structure search on the ChemSpider database of the Royal Society of Chemistry.

## Data Availability

The authors confirm that the data supporting the findings of this study are available within the article.

## Supplementary Materials

The following are available online at https://www.mdpi.com/article/10.3390/ijms22158320/s1, Table S1: List of compounds selected from commercial chemical libraries for the aptamer-based competitive drug screening assay designed in this study.
