# Supplementary material for: Novel Aptamer-Based Small-Molecule Drug Screening Assay to Identify Potential Sclerostin Inhibitors against Osteoporosis
Source: Int J Mol Sci. 2021 Aug 2;22(15):8320. doi: 10.3390/ijms22158320 (PMC8348959; doi:10.3390/ijms22158320)
Supplement: Supplementary file 1 [file ijms-22-08320-s001.zip › ijms-1234809-supplementary.pdf]

Table S1 List of compounds selected from commercial chemical libraries for the aptamer-based competitive drug screening assay designed in this study

Product name	Molecular weight	CAS number	Formula	Target
**Compounds containing at least one tertiary amino group**				
Methotrexate*	454.44	59-05-2	$C_{20}H_{22}N_8O_5$	Dihydrofolate reductase
Ketoconazole*	531.43	65277-42-1	$C_{26}H_{28}Cl_2N_4O_4$	Cytochrome P450
Sildenafil citrate*	666.7	171599-83-0	$C_{28}H_{38}N_6O_{11}S$	Phosphodiesterase
Mycophenolate mofetil*	433.49	128794-94-5	$C_{23}H_{31}NO_7$	Inosine monophosphate dehydrogenase
Dyphylline*	254.24	479-18-5	$C_{10}H_{14}N_4O_4$	Phosphodiesterase
Tadalafil*	389.4	171596-29-5	$C_{22}H_{19}N_3O_4$	Phosphodiesterase
Apixaban*	459.5	503612-47-3	$C_{25}H_{25}N_5O_4$	Factor Xa
Aminophylline*	420.43	317-34-0	$C_{16}H_{24}N_{10}O_4$	Phosphodiesterase
Fluvastatin sodium*	433.45	93957-55-2	$C_{24}H_{25}FNNaO_4$	HMG-CoA Reductase
Atorvastatin calcium*	1155.34	134523-03-8	$C_{66}H_{68}CaF_2N_4O_{10}$	HMG-CoA Reductase
Ozagrel*	228.25	82571-53-7	$C_{13}H_{12}N_2O_2$	Factor Xa
Vardenafil HCl trihydrate*	579.11	224785-90-4	$C_{23}H_{39}ClN_6O_7S$	Phosphodiesterase
Clarithromycin*	747.95	81103-11-9	$C_{38}H_{69}NO_{13}$	Cytochrome P450
Cobicistat (GS-9350)*	776.02	1004316-88-4	$C_{40}H_{53}N_7O_5S_2$	Cytochrome P450 (e.g. CYP17)
Rivaroxaban*	435.88	366789-02-8	$C_{19}H_{18}ClN_3O_5S$	Factor Xa
Anagrelide HCl*	292.55	58579-51-4	$C_{10}H_8Cl_3N_3O$	Phosphodiesterase
Moclobemide (Ro 111163)*	268.74	71320-77-9	$C_{13}H_{17}ClN_2O_2$	Monoamine oxidase
Avanafil*	483.95	330784-47-9	$C_{23}H_{26}ClN_7O_3$	Phosphodiesterase
GSK256066	518.58	801312-28-7	$C_{27}H_{26}N_4O_5S$	Phosphodiesterase
PF-3845	456.46	1196109-52-0	$C_{24}H_{23}F_3N_4O_2$	Fatty acid amide hydrolase
Nepicastat (SYN-117) HCl	331.81	170151-24-3	$C_{14}H_{16}ClF_2N_3S$	Hydroxylase
CP-91149	399.87	186392-40-5	$C_{21}H_{22}ClN_3O_3$	Phosphorylase
Torcetrapib	600.47	262352-17-0	$C_{26}H_{25}F_9N_2O_4$	Cholesterol ester transfer protein
Lonafarnib	638.82	193275-84-2	$C_{27}H_{31}Br_2ClN_4O_2$	Farnesyltransferase
JNJ-1661010	365.45	681136-29-8	$C_{19}H_{19}N_5OS$	Fatty acid amide hydrolase
PF-4981517	456.59	1390637-82-7	$C_{26}H_{32}N_8$	Cytochrome P450

Product name	Molecular weight	CAS number	Formula	Target
Evacetrapib (LY2484595)	638.65	1186486-62-3	$C_{31}H_{36}F_6N_6O_2$	Cholesterol ester transfer protein
(*R*)-Nepicastat HCl	331.81	195881-94-8	$C_{14}H_{16}ClF_2N_3S$	Hydroxylase
NLG919	282.38	1402836-58-1	$C_{18}H_{22}N_2O$	Indoleamine 2,3-dioxygenase
AGI-5198	462.56	1355326-35-0	$C_{27}H_{31}FN_4O_2$	Dehydrogenase
PF-04620110	396.44	1109276-89-2	$C_{21}H_{24}N_4O_4$	Transferase
Deltarasin	603.75	1440898-61-2	$C_{40}H_{37}N_5O$	Phosphodiesterase
Erastin	547.04	571203-78-6	$C_{30}H_{31}ClN_4O_4$	Ferroptosis
Edoxaban	548.06	1229194-11-9	$C_{31}H_{40}ClN_7O_8S_2$	Factor Xa
LB42708	555.46	226929-39-1	$C_{30}H_{27}BrN_4O_2$	Transferase
Darapladib (SB-480848)	666.77	356057-34-6	$C_{36}H_{38}F_4N_4O_2S$	Phospholipase (e.g. PLA)
U73122	464.64	112648-68-7	$C_{29}H_{40}N_2O_3$	Phospholipase (e.g. PLA)
Apremilast (CC-10004)	460.5	608141-41-9	$C_{22}H_{24}N_2O_7S$	Phosphodiesterase
Fostamatinib (R788)	580.46	901119-35-5	$C_{23}H_{26}FN_6O_9P$	Spleen tyrosine kinase
AP26113	529.01	1197958-12-5	$C_{26}H_{34}ClN_6O_2P$	Anaplastic lymphoma kinase
GDC-0941	513.64	957054-30-7	$C_{23}H_{27}N_7O_3S_2$	Phosphoinositide 3-kinase
Tipifarnib	489.4	192185-72-1	$C_{27}H_{22}Cl_2N_4O$	Farnesyltransferase, Ras
**Compounds containing primary and/or secondary but without tertiary amino groups**				
Cilostazol*	369.46	73963-72-1	$C_{20}H_{27}N_5O_2$	Phosphodiesterase
Rolipram*	275.34	61413-54-5	$C_{16}H_{21}NO_3$	Phosphodiesterase
Topiramate*	339.36	97240-79-4	$C_{12}H_{21}NO_8S$	Carbonic anhydrase
Pralatrexate*	477.47	146464-95-1	$C_{23}H_{23}N_7O_5$	Dihydrofolate reductase
Pimobendan*	334.37	74150-27-9	$C_{19}H_{18}N_4O_2$	Phosphodiesterase
Irsogladine*	256.09	57381-26-7	$C_9H_7Cl_2N_5$	Phosphodiesterase
Pyrimethamine*	248.71	58-14-0	$C_{12}H_{13}ClN_4$	Dihydrofolate reductase
Pioglitazone HCl*	392.9	112529-15-4	$C_{19}H_{21}ClN_2O_3S$	Peroxisome proliferator-activated receptor
Roflumilast*	403.21	162401-32-3	$C_{17}H_{14}Cl_2F_2N_2O_3$	Phosphodiesterase
Milrinone*	211.22	78415-72-2	$C_{12}H_9N_3O$	ATPase
Brinzolamide*	383.51	138890-62-7	$C_{12}H_{21}N_3O_5S_3$	Carbonic anhydrase
Methazolamide*	236.27	554-57-4	$C_5H_8N_4O_3S_2$	Carbonic anhydrase
Gimeracil*	145.54	103766-25-2	$C_5H_4ClNO_2$	Dehydrogenase

Product name	Molecular weight	CAS number	Formula	Target
Rasagiline mesylate*	267.34	161735-79-1	$C_{13}H_{17}NO_3S$	Monoamine oxidase
S-(+)-Rolipram*	275.34	85416-73-5	$C_{16}H_{21}NO_3$	Phosphodiesterase
Phosphoramidon disodium salt	588.48	164204-38-0	$C_{23}H_{34}N_3Na_2O_{10}P$	Metalloendopeptidase
Safinamide mesylate	398.45	202825-46-5	$C_{18}H_{23}FN_2O_5S$	Monoamine oxidase
Liproxstatin-1	340.85	950455-15-9	$C_{19}H_{21}ClN_4$	Ferroptosis
Epacadostat (INCB024360)	438.23	1204669-58-8	$C_{11}H_{13}BrFN_7O_4S$	Indoleamine 2,3-dioxygenase
GSK3787	392.78	188591-46-0	$C_{15}H_{12}ClF_3N_2O_3S$	Peroxisome proliferator-activated receptor
WY-14643 (Pirinixic acid)	323.8	50892-23-4	$C_{14}H_{14}ClN_3O_2S$	Peroxisome proliferator-activated receptor
PluriSIn #1 (NSC 14613)	213.24	91396-88-2	$C_{12}H_{11}N_3O$	Dehydrogenase
TAK-700 (Orteronel)	307.35	566939-85-3	$C_{18}H_{17}N_3O_2$	Cytochrome P450
Telotristat etiprate (LX 1606 Hippurate)	754.15	11137608-69-5	$C_{36}H_{35}ClF_3N_7O_6$	Hydroxylase
UK 383367	324.38	348622-88-8	$C_{15}H_{24}N_4O_4$	Procollagen C proteinase
INCB024360	271.64	914471-09-3	$C_9H_7ClFN_5O_2$	Indoleamine 2,3-dioxygenase
URB597	338.4	546141-08-6	$C_{20}H_{22}N_2O_3$	Fatty acid amide hydrolase
A922500	428.48	959122-11-3	$C_{26}H_{24}N_2O_4$	Diacylglycerol Acyltransferase 1
Dalcetrapib (JTT-705, RO4607381)	389.59	211513-37-0	$C_{23}H_{35}NO_2S$	Cholesterol ester transfer protein
AGI-6780	481.51	1432660-47-3	$C_{21}H_{18}F_3N_3O_3S_2$	Dehydrogenase
Ferrostatin-1 (Fer-1)	262.35	347174-05-4	$C_{15}H_{22}N_2O_2$	Ferroptosis
Vidofludimus	355.36	717824-30-1	$C_{20}H_{18}FNO_4$	Dehydrogenase
FTI 277 HCl	484.07	180977-34-8	$C_{22}H_{30}ClN_3O_3S_2$	Transferase
D4476	398.41	301836-43-1	$C_{23}H_{18}N_4O_3$	Casein kinase 1
U-104	309.32	178606-66-1	$C_{13}H_{12}FN_3O_3S$	Carbonic anhydrase
**Compounds with no amino group**				
Voriconazole*	349.31	137234-62-9	$C_{16}H_{14}F_3N_5O$	Cytochrome P450
Isotretinoin*	300.44	4759-48-2	$C_{20}H_{28}O_2$	Hydroxylase
Trilostane*	329.43	13647-35-3	$C_{20}H_{27}NO_3$	Dehydrogenase
Lovastatin*	404.54	75330-75-5	$C_{24}H_{36}O_5$	HMG-CoA reductase

Product name	Molecular weight	CAS number	Formula	Target
Tioxolone*	168.17	4991-65-5	$C_7H_4O_3S$	Carbonic anhydrase
Pravastatin sodium*	446.51	81131-70-6	$C_{23}H_{35}NaO_7$	HMG-CoA reductase
Tolcapone*	273.24	134308-13-7	$C_{14}H_{11}NO_5$	Transferase
Avasimibe	501.72	166518-60-1	$C_{29}H_{43}NO_4S$	Cytochrome P450
PF-2545920	392.45	1292799-56-4	$C_{25}H_{20}N_4O$	Phosphodiesterase
GW0742	471.49	317318-84-6	$C_{21}H_{17}F_4NO_3S_2$	Peroxisome proliferator-activated receptor
Cilomilast	343.42	153259-65-5	$C_{20}H_{25}NO_4$	Phosphodiesterase
Apigenin	270.24	520-36-5	$C_{15}H_{10}O_5$	Cytochrome P450
Baicalein	270.24	491-67-8	$C_{15}H_{10}O_5$	Cytochrome P450
Icariin	676.66	489-32-7	$C_{33}H_{40}O_{15}$	Phosphodiesterase
Luteolin	286.24	491-70-3	$C_{15}H_{10}O_6$	Phosphodiesterase
Naringenin	272.25	480-41-1	$C_{15}H_{12}O_5$	Cytochrome P450
Alizarin	240.21	72-48-0	$C_{14}H_8O_4$	Cytochrome P450
CPI-613	388.59	95809-78-2	$C_{22}H_{28}O_2S_2$	Dehydrogenase
Xanthohumol	354.4	6754-58-1	$C_{21}H_{22}O_5$	Cyclooxygenase

*FDA approved drugs
